# Hyperemesis Gravidarum in the context of migration: when the absence of cultural meaning gives rise to “blaming the victim”

**DOI:** 10.1186/s12884-019-2344-1

**Published:** 2019-06-10

**Authors:** Danielle Groleau, Jessica Benady-Chorney, Alexandra Panaitoiu, Vania Jimenez

**Affiliations:** 1 Division of Social and Transcultural Psychiatry, McGill University; Culture and Mental Health Unit - Lady Davis Medical Institute, Jewish General Hospital- CIUSSS West-Central Montreal, 4333 Chemin de la Côte-Ste-Catherine, Montréal, QC H3T 1E4 Canada; 2 Department of Psychiatry, University of Toronto, 500 University Avenue, Suite 602, Toronto, ON M5G 1V7 Canada; 3 Department of Family Medicine, McGill University, CLSC de Côte-des-Neiges- CIUSSS West-Central Montreal, 5700 Chemin de la Cote-des-Neiges, Montreal, QC H3T 2A8 Canada

**Keywords:** Hyperemesis gravidarum, Depression, Migration, Access to care, “blaming the victim,” culture

## Abstract

**Background:**

Hyperemesis gravidarum (HG) is a rare complication of pregnancy that involves persistent nausea and extreme vomiting to an intensity that differentiates HG from nausea and vomiting commonly experienced during pregnancy. Research has suggested potential biological and psychological etiological pathways for HG, but the augmented prevalence in immigrant populations, which is 4.5 times higher, remains unclear. Studies show that in order to better address the psychosocial needs of immigrant patients with HG, we must first improve our understanding of how they experience their illness. The objective of this study was to understand the meaning and experience of HG among immigrant women in Canada.

**Methods:**

Our “qualitative comparative analysis design” involved a sample of 15 pregnant mothers following their hospitalization for HG, including 11 immigrant women and 4 Canadian-born women recruited for comparison purposes. We used the Edinburgh Perinatal Depression Scale to assess distress, and the McGill Illness Narrative Interview Schedule to explore how pregnant women understood and experienced their HG and the health services that they received.

**Results:**

With the exception of a few women whose mothers suffered from HG, immigrant women and their loved ones did not have cultural knowledge to attribute meaning to HG symptoms. This left them vulnerable to criticism from family, as well as feelings of self-doubt, stress, and anxiety. We interpret this phenomenon as ‘victim blaming’. Immigrant women’s experience of HG was also characterised by high levels of depressive symptoms (40%) which they linked to the severity of their symptoms, high levels of stress associated with adapting to their new country, social isolation, and loss of female family members. Furthermore, in contrast to Canadian-born women, immigrant women frequently reported feeling that their symptoms were minimized by hospital emergency room medical staff, which led to delays in obtaining appropriate health care. However, once admitted to hospital, they perceived the care provided by dieticians and nurses as helpful in managing their symptoms.

**Conclusions:**

Wider awareness of the impact of HG may improve the quality of family support for immigrant women. There is a need to improve the delays and appropriateness of clinical care.

## Background

The severity of nausea and vomiting in pregnancy ranges as a continuum from the nausea and vomiting that occurs commonly in many pregnancies to hyperemesis gravidarum (HG), a rare and severe complication involving persistent nausea and extreme vomiting associated with ketosis and weight loss (> 5% of pre-pregnancy weight). HG leads to nutritional deficiencies, loss of water and salts from cells, and acid-base imbalances that can cause destabilised organ function, renal failure, neurological damage, and even death [1]. The severity and persistence of HG symptoms, which can endanger the health of both mother and child, differentiate HG from the nausea and vomiting commonly experienced by women during pregnancy.

HG is a rare disorder estimated to affect between 0.03–2% of pregnancies [2–4], with a prevalence up to 4.5 times higher in immigrant populations [5–8]. Country of origin and length of time after immigration were shown to influence this prevalence [9], with only one study in China reporting a rate as high as 10.8% in pregnant immigrant women [10]. Although research has suggested potential biological and psychological etiological pathways for HG, it remains unclear why the prevalence of this illness is so much higher in immigrant women.

### Biological factors

Research has identified potential hormonal, immunological, gastrointestinal, and genetic causes of HG. However, the extent and way in which specific biological factors, such as estrogens, human chorionic gonadotropin, prolactin, cortisol, leptin, progesterone, and thyroid hormones play a role in the etiology of HG is still under debate, with studies finding both increases and decreases in each of the biological measures [11, 12]. Similarly, the relationship between helicobacter pylori infection and HG remains controversial, with some studies suggesting a strong association [13, 14] and others suggesting that the relationship is much weaker [11, 12, 15]. Recently, the risk of HG has also been shown to be influenced by maternal genotype [16, 17] and was linked to genes mediating calcium channels that are activated through thyroid hormone signalling [18]. However, these biological factors do not explain why immigrant women are more vulnerable to developing HG.

### Psychological factors

Initial interest in psychological determinants of HG was manifested by studies that claimed that HG was a form of somatisation involving a maternal rejection of the foetus [19]. More recent studies on the psychological dimensions of HG have associated HG with symptoms of depression, anxiety [20–22], and post-traumatic stress disorder [23]. However, these more recent studies suggest that psychological distress may be a reaction to the severity of HG symptoms rather than a central part of its etiological pathways [24–26]. Nevertheless, authors do agree that there is a need for more studies to better understand the complex role of psychological factors in HG by exploring women’s illness meaning and experience in relation to distress [27].

### Immigrant women

The higher prevalence of HG in the immigrant population adds a layer of complexity to the problem. Whether the potential biological and psychological causal factors of HG suggested for the general population could explain the higher prevalence of HG in immigrant women remains unresolved in the literature [5, 7–9, 28, 29]. Elevated rates of HG among immigrants do raise the possibility that factors related to experiencing pregnancy during the migration process could mediate other psychological and corresponding biological factors associated with HG [5].

There is conflicting evidence regarding whether immigrant women in good physical health are at greater risk for mental health problems during the perinatal period [30, 31]. On one hand, population-based health surveys in Canada support a healthy immigrant effect, with immigrants in general having better mental health at the time of migration but gradually worsening mental health over time [31]. However, a few studies have found that immigrant women are more vulnerable during reproductive periods, including being four times more likely than non-immigrant women to present with depression during pregnancy [32] and twice more likely to develop non-psychoticpost-natal depression [33, 34].

The aforementioned studies do not allow to establish valid conclusions about the causes of the higher rates of HG in immigrant populations. This points to the need for exploratory studies on the meaning and experience of this serious condition from the perspective of immigrant women themselves, in order to better understand the underlying psychological and social processes related to HG. Our study therefore aimed to explore the illness meaning and experience of HG among immigrant pregnant women.

## Methods

### Recruitment and sample

We recruited patients over a 10-month period at two hospitals located in a highly multicultural neighbourhood in Montreal (Quebec, Canada). Initial inclusion criteria for the participants were as follows: being 18 years of age or older, being born outside Canada, having received an HG diagnosis [1], having been prescribed anti-emetic drugs, and having been hospitalised because of HG in one of two participating hospitals. Because of the expected scarcity of HG cases, we did not use any other exclusion criteria and accepted all eligible mothers regardless of their country of origin. All mothers invited to participate in the study accepted to take part. Despite the fact that the immigrant women recruited came from various cultural backgrounds, we rapidly obtained saturation of the data necessary to answer our research question (after seven interviews) because the focus of the women’s narratives addressed mainly issues of migration and the severity of their symptoms. After saturation was attained with immigrant mothers we decided to also recruit among Canadian-born mothers using the same clinical criteria, in hopes that comparison of their experience of HG to that of immigrant women would help shed light on the role of migration in the HG experience. We proceeded to recruit additional participants including four pregnant immigrant women and four Canadian-born women. As we did not have a sufficient number of Canadian-born mothers to constitute a formal case-study design [35], our “qualitative comparative analysis design” (QCA) [36] thus involved a total sample of 15 pregnant mothers (*n* = 15) following their hospitalisation for HG, including 11 immigrant women and 4 Canadian-born women that we compared to each other. Due to the extreme scarcity of HG cases and the fact that we rapidly obtained saturation with immigrant patients, we considered that the QCA was an optimal design to shed theoretical light on the experience of HG in the context of migration [36].

### Measures

We used the McGill Illness Narrative Interview Schedule (MINI) [37] to explore the illness meaning and experience of HG with immigrant and Canadian-born participants. We also used a short socio-demographic questionnaire for descriptive purposes as it is very common in qualitative designs. Given the uncertainty around the role of distress in the aetiology of HG [36], we also used the Edinburgh Postnatal Depression Scale (EPDS) to provide a picture of the distress level of our participants alongside their socio-demographic characteristics.

The EPDS is a 10-itemself-report screening questionnaire designed to rapidly screen for depressive symptoms in pregnancy and the post-partum period [38]. The EPDS has been validated in multiple languages [39] and was therefore an optimal choice given the multiple linguistic backgrounds of our participants.

The MINI is a generic semi-structured ethnographic interview schedule translated into 13 languages and designed to explore illness meaning and experience in a cultural context, corresponding health behaviours, pathways to care, and changes in world view following an illness [37]. The structured dimension of the MINI allowed us to compare the illness meaning and experience of immigrant women with that of Canadian-born women. The MINI builds on the theoretical argument of Young [40], stating that while patients reason in a causal way when they produce attributions for their illness when using explanatory models of illness, they can also reason analogically when comparing their illness to past prototypical illness experiences of self or others. Young [40] also argues that when ill, people can reason in a metonymic way when they produce illness narratives that connect past events such that they link temporally to the actual illness. The MINI is constructed to elicit the complexity of meaning expressed within these three modes of reasoning (causal, analogical, metonymic), using three different forms of knowledge structure (explanatory models, prototypes, illness narratives). In this way, interviews using the MINI can reveal the complexity of illness meaning and experience within historical, social, and cultural contexts.

The MINI interviews lasted one hour on average and were all conducted by the same research assistant following participants’ hospital discharge. While interpreters were available to all participants, interviewees did not request them. The first part of the MINI interview was relatively unstructured and aimed to collect the narrative of participants’ migration to Canada, their pregnancy and their illness starting with the presentation of their first symptoms. This enabled the understanding of their metonymical reasoning and the context of their HG experience. Examples of some of the questions posed were: “Let’s start with the story of your migration. How did you decide to come here to Canada?”; “When did you discover you were pregnant?”; “Could you tell us when you realized you had this HG problem?”; “When did it become excessive?”

The next part of the interview included structured open-ended questions that invited participants to elicit illness prototypes about self and others and compare their current HG condition to these illness prototypes. Examples of some questions posed were: “In the past, have you ever had a health problem that you consider similar to your actual HG? In what way is that past health problem similar or different to your actual HG?”; “Did a person in your family or social environment experience a health problem similar to your HG? If yes, how do you consider your HG to be different or similar to this other person’s health problem?”

Following the section on illness prototypes, there was a third structured section with questions aiming to elicit the explanatory models of participants’ HG (e.g., perceived causes, cultural labels and expected outcomes based on their cultural knowledge) in order to identify if and how the attributions they gave to their illness were linked to a cultural theory of health and illness. Community studies have indeed shown that past prototypical illness experiences and explanatory models are not always idiosyncratic but often refer to local popular theories of health and illness that can influence the adoption of health behaviours and may reflect the underpinnings of the sociocultural context of the narrator [41]. Examples of questions asked in this section were: “According to you, what caused your HG?”; “Do people close to you have ideas about what could cause your health problem?”; “Why did your HG start at the precise moment it did?”

The fourth part of the MINI interview explored the patients’ narratives of their pathways to care within the health system (e.g. clinic, hospital) but also within informal care (e.g., family, healers, alternative medicine). Examples of questions posed in this section were: “What did you do to try to alleviate your symptoms linked to HG?”; “Did you go to see someone for help like a healer, a herb doctor, or alternative medicine practitioner?”

The fifth section of the MINI explored changes in the participants’ worldview since their experience of HG. In the case of this study, this included the way participants perceived their current and future pregnancies. The following are examples of the questions posed: “Can you explain what it meant for you to have HG?”; “Did your HG change the way you perceive being pregnant in general?”; “Has your HG changed your way of viewing your baby?”

Finally, we ended with a section on sources of support. Examples of some of the questions posed were: “What aspect of your life helped you go through this difficult period? Could you explain in what way? Who helped you the most while you had this problem?”

### Procedure

Women with a diagnosis of HG were approached by clinical staff during their hospital stay to obtain permission to receive a phone call after their discharge from a research assistant who would explain the study and invite them to participate. Ethical approval was obtained by each of the two participating hospitals’ ethical review boards. The participating pregnant women were interviewed at their homes shortly after their hospital discharge. The socio-demographic questionnaire and EPDS were also completed during the home visit. Trained medical interpreters from the Regional Health Board of Montreal were made available to participating mothers but none of the mothers felt it was necessary to use them. However, while none of the women required a translator, during a few interviews, the husbands intervened to clarify or elaborate certain ideas put forward by the pregnant women.

### Data analysis

The socio-demographic data and EPDS were compiled to calculate frequencies for descriptive purposes only. Interviews were audio recorded, transcribed, and transferred to Atlas-ti for qualitative coding and analysis following a “middle range analysis” using two types of codes: conceptual and thematic [42]. Coding was completed by the second author under the supervision of the first author. We started by creating a predetermined list of conceptual codes that we used deductively to code the sections of the interviews that reflected the theoretical concepts underlying the structure of the MINI (e.g., prototypes of self, prototypes of others, explanatory models, events linked to HG, experience of health services, changes in worldview, view of pregnancy). We also applied inductive coding by creating thematic codes as they emerged from the transcribed interviews. The 75 thematic codes produced were then clustered by the first author, with the help of the second author, into 45 larger categories of themes based on meaning. We separated the sample into two sub-samples (immigrants vs. Canadian-born) and created separate summaries for these two groups of mothers for larger categories of thematic codes as well as for conceptual codes. Summary of the immigrant mother group were then compared to the corresponding summary of Canadian-born mothers to verify whether their illness meanings and experiences were similar or different and to explore whether these differences may have been linked to the migration process. The first author then used each summary to answer the research question, taking into consideration the differences between the two sub-samples.

The first version of the analysis was discussed with the second, third and last authors. For reflexivity purposes, we state that none of the authors had biases during the interpretation of the results. However, the first author, who is Canadian-born, had first-hand experience of HG during her first pregnancy, but was not hospitalised, did not take medication, and did not experience the phenomenon of “victim blaming” prevalent among the immigrant participants in this study. The results section will present a synthesis of the results, and the discussion section will present our interpretation of these results in light of the research question and the empirical and conceptual literature.

## Results

### Description of the sample

As seen in Table 1, the majority of participants were in their twenties (9/15) and lived with a partner (11/15). Just over half of the participants (8/15) were first-time mothers, with (6/11) having arrived in Canada within the past 5 years.

**Table 1:** EPDS score and socio-demographic characteristics of 15 participants

	n	%
EPDS score suggesting probable depression (average EPDS score of 21/30 with remaining others average score of 9/30)	6	40
Age		
20–25	4	27
25–30	5	33
30–35	6	40
Country of birth		
Lebanon	1	6.7
Cameroon	1	6.7
Syria	1	6.7
Venezuela	1	6.7
Morocco	2	13.3
Congo	1	6.7
Philippines	2	13.3
Jamaica	1	6.7
Sri Lanka	1	6.7
Canada	4	26.7
Number of children		
0	8	53.3
1	4	26.7
2	1	6.7
3	2	13.3
Civil status		
Married	10	66.7
Single	3	20
Common-law union	1	6.7
Separated	1	6.7
Education level		
High School (Grade 11)	4	26.7
CEGEP	2	13.3
Diploma of Vocational Studies	1	6.7
Bachelor’s	7	46.7
Master’s	1	6.7
Employment Status		
Unemployed	6	40
Employed	9	60
Language at home		
English	5	33.5
French	4	26.7
Arabic	3	20
Tagalog	2	13.3
Tamil	1	6.7
Current status in Canada		
Permanent resident	7	46.7
Canadian citizen	8	53.3
No. of relatives in Canada		
0	4	26.7
1	2	13.3
4	2	13.3
5	1	6.7
> 5	4	26.7
Information unavailable	2	13.3
No. of live-in relatives		
0	10	66.7
1	2	13.3
3	1	6.7
4	1	6.7
Information unavailable	1	6.7
Length of residence in Canada		
N/A (non-immigrant)	4	27
5 years or more	6	40
Less than 5 years	5	33

In the following results section, we will highlight the characteristics of Canadian-born mothers only when they differ from those of immigrant mothers.

### Cultural knowledge of HG

Among the immigrant women interviewed, there was a marked absence of cultural knowledge and explanatory models for HG. In contrast, most Canadian-born women (3/4) attributed their HG to hormones (2/3) and to stress and anxiety (1/3). Very few immigrant women had illness prototypes relating to their HG, in contrast to Canadian-born women (3/4), who either had a family member with a previous experience of HG or saw themselves as generally vulnerable to nausea before they became pregnant. A total of 9/15 patients had never heard of HG before their illness and were informed of it for the first time when they were admitted to hospital. Among these, close to half (4/9)—including only one Canadian-born mother—reported that their loved ones had downplayed their symptoms or accused them of exaggerating, often mistaking their symptoms for the nausea typically seen in the first trimester of pregnancy:

**611:** “When I explain this to people, they don’t get it. There are some friends who don’t get it because, with them, it works. It can make you feel a bit … a bit judged or in any case not really understood in terms of what you’re going through. Also, my husband sometimes doesn’t believe me. Like last time I was hospitalized he said ‘It’s nothing’ … and when I was admitted for long-term stay, that’s when he realized ‘Ah ok.’”

These patients felt self-doubt regarding the legitimacy of their illness:

**609:** “We have a tendency … I don’t know for you but for me, I have a tendency when I see that everyone is watching me, like my mother in France: ‘You’re exaggerating!’ It’s not that I’m exaggerating. We have a tendency to refer to our elders to try and make sense of things. Everyone tells you: ‘Me too, when I was pregnant, I felt that. It’s normal. You’ll see. At your fourth month it’ll be much better. I say ‘Oh Okay! Everyone has felt the same thing … it’s normal!’ But it’s just the time that I come to the emergency for the first time … I see my doctor … stop working... go to the emergency a second time. At that point I told myself [laughs] this isn’t normal! Everyone I think thought the same thing—I was exaggerating in a way. Maybe it’s me who is weak. Maybe I’m not a patient … maybe I’m amplifying things.”

A lack of cultural explanations for HG symptoms thus left many immigrant women vulnerable to criticism and self-doubt. Conversely, women who mentioned HG illness prototypes during the interview were less likely to experience criticism from family members or friends. Here are examples of an immigrant woman and a Canadian-born woman referring to their mothers’ HG prototype:

**603:** “My mother told me when she was pregnant with me—she told me she had exactly the same thing, she was even hospitalized the last 4 months of her pregnancy. I saw in Cameroon people who vomited a lot, they weren’t exaggerating either. I received a lot of support from my family.”

**731:** “My mother, I think she lost more weight than me. She told me that she lost over 15 pounds. She was at 90 pounds. Only when she was pregnant. I find it’s a problem with me that when I get pregnant and that will pass after that. After the 9 months, it will go and I won’t have it anymore”

The above excerpts demonstrate how having an HG prototype protected women from feelings of self-doubt and insecurity, a situation that makes sense if one knows others who have this type of extreme experience.

### Experience of HG

Patient narratives also linked the HG experience to events related to high levels of anxiety, challenges in adapting to their new environments, loss of family members, and loss of the desire to be pregnant due to the severity of their symptoms. In close to half of the cases, (*n* = 8), HG symptoms appeared around a time when patients experienced high levels of stress or anxiety. This makes sense in the light of the fact that 40% of the mothers of our sample scored above the threshold for depression on the EPDS scale. Correspondingly, some mothers directly associated their symptoms with feelings of social isolation. One patient’s husband, who added to his wife’s narrative, gave the following account:

**604:** “She's here by herself. Her mother is in Syria, her sister, everybody, she' s here by herself. She has relatives but not that close. Maybe this loneliness also affected her, you know, symptoms and they worsen. You know, when you have … when you're homesick you feel everything is (ugly).”

Isolation may also lead some mothers to focus more on their bodily symptoms, as illustrated in this excerpt:

**609**: “Sometimes I think it might be psychological as well. I think I’m going to vomit all the time and then I vomit. I tell myself, in half an hour, I’ll have to vomit … it’s like at a certain time, I’ll have to vomit … with someone with me, we’re chatting and I forget about it … sometimes I’m lying down and trying to sleep and I get up thinking I’m going to vomit in a few minutes. Sometimes, it happens; I vomit because I’m thinking about it.”

Some mothers felt weak and helpless because of their anxiety:

**603:** “I tell myself that it’s linked to anxiety, and after two months we ask ourselves ‘Will I be capable? Do I have the power?’**”**

**619:** "I could hardly talk on the phone, hardly do anything … I got very anxious during that time about everything. Things that weren’t even important or that I shouldn’t have been anxious about … I had the support I needed from everyone, but one night I remember at the hospital I stayed awake wondering if there’s a fire in the hospital and would I have enough energy to get out."

Other immigrant mothers, as well as half of the Canadian-born mothers, noted that they became ill following disruptions, such as moving, adapting to life in Canada or to a new environment, or loss of a family member:

**601:** “It was a month before I got pregnant that my father died … I was here, yes. I couldn't go down there. I think I had a lot of stress in the first and second month … my father … [the] new environment and [being] away from my family. But also I don't know if it's stress or other feelings too, together. My family [if they were here] maybe it would be easier for me.”

**616:** “The only thing that happened before that was the moving. I can’t see what else it could be. I regret it a bit … But you can’t really change that. It’s what I wanted to do. One could say that I felt more at home there, but like they say it takes time to adapt.”

The above excerpts point to the impact of changing environments on the mothers’ HG symptoms. These changes in environment may have also contributed to feelings of isolation and unfamiliarity.

While 40% of the patients (a majority being recent immigrants) mentioned suffering significantly from missing one or more family members during the time they were ill, it was rare for patients to spontaneously attribute their HG to missing family members**.** Half of Canadian-born mothers also mentioned missing either a mother or a sister who lived far away. However, all patients whose mothers or mothers-in-law were present during their illness commented on the benefits of having their support:

**609**: “She’s a mother hen because she knows that *I* don’t have a tendency to exaggerate. She says ‘Okay, surely there’s a reason, the fact that she’s like this.’ So she was there for me the whole time. She came with me to the emergency room. She practically never left my side.”

In contrast, participants whose mothers were not present commented on the anguish they felt from missing their mothers while ill. On being asked whom they missed the most, these women usually indicated that it was their mothers:

**601:** “My mother and sister … they'd help me more with the house … someone to be with me, if I need anything.”

**611:** "My mother … she would’ve been there for me, she would’ve helped me by providing me with affection, helping me take care of the kids, etc."

One participant whose mother had also suffered from HG responded:

**603:** "I missed my mother because when you experience certain things, and you come to learn what they must have experienced, it heightens your admiration and love for that person and you just want to share that with them, and she’s so far … It would be different I don’t know … Because we talk on the phone but it’s not enough. By talking with her, listening, sharing, and comparing our experiences, yeah I think it would have made things easier for me [to have her here]."

A total of 80% (*n* = 12) of participants felt that their HG prevented them from enjoying their pregnancy and interfered either with bonding with their unborn child or with maintaining their maternal role with their other children:

**621:** "It's only when I'm pregnant that I vomit. So like the perception of being pregnant is like … if it's like this, it's no good. Even if there's help, there's medication, there's things like that, you vomit anyway. You feel like being pregnant. You're happy because you're carrying something, you look forward to, but no, you're focusing on your vomiting, you're sad, you're like this, you know you don't enjoy it. Usually, you enjoy the fact that oh my god, I'm pregnant! It's hard to be pregnant. So the way you look at it it's supposed to be happy, not to make you miserable."

**609**: “I didn’t envision this about pregnancy. [It was] totally different. I always thought that pregnancy could be one of the most beautiful moments that happen. Especially when you’re all well in your head, you have a good entourage, you don’t have any problems, no health problems, but … since then I’ve realised that’s not necessarily true.”

In some cases, the severity of the symptoms drove patients to consider abortion or deterred them from having other children:

**621**: “I don't want to be pregnant any more, because of the experience I had with my first one. The first one I had this vomiting until I had the baby … You know, like when I found out that I was pregnant, Oh my God, not again! You mean I'm going to have this for 9 months!”

**617**: "I'm still happy that I'm pregnant. I'm still happy that I'm pregnant, but I just wish it wasn't like this... Honestly I don't want to get pregnant again."

Despite this, participants stated that ultimately, their suffering did not change the way they viewed their unborn child:

**621**: “Although sometimes like ah no, I'm pregnant again … It's not his fault, that damn vomiting, it's just me.”

**625**: “I think I’m growing closer to the baby. Because I guess the more you suffer, the more love you give to the child, you know?”

### Experience of clinical care

Immigrant participants felt that their symptoms were not always taken seriously by medical staff when they presented to the hospital emergency room. Often, their HG symptoms were misinterpreted as either being the nausea and vomiting associated with normal pregnancy or some other illness:

**604:** “The doctor checked on me and in fact they suspected something else, they thought it was my appendix.”

**609: “**I was vomiting a lot, a lot … I had gone to see the doctor and she told me I had to rest, no stress, that she would follow me. She told me ‘If ever it worsens, don’t hesitate. Go to the hospital’. So, in the first month, I arrived at the hospital at 9:30 in the morning and I was back at my place around 7:00 pm. They told me ‘Okay it’s not bad. It’s a pregnancy. Normal, it happens.’ They ran tests, everything was fine. They told me ‘It’s rest … you can go back to your place.’ Then they saw me at work and told me ‘Impossible!’ They sent me home and I returned to see the doctor. I came to the hospital and they kept me. There were some who hesitated; they wanted to send me back home while others wanted to keep me—they didn’t know what to do until a resident came and she said she had seen the results. She told me that according to the test I was really dehydrated. So... impossible, they’re keeping me! A really unpleasant experience.”

The above excerpts demonstrate examples of clinical misinterpretation of HG symptoms, which resulted in delayed diagnosis and treatment for some mothers. However, the Canadian-born mothers did not mention having this problem, indicating that they received proper attention either when presenting to the hospital emergency room (3/4) or while being admitted directly to the ward (1/4).

With regard to satisfaction with clinical care, all patients mentioned appreciating the care they received from nurses and dieticians:

**608**: “The nurses, very good. It’s just I found it hard (we see the doctor) one time per day. Sometimes, I had questions, I didn’t feel well, nurses couldn’t give me answers. So that’s it, it’s just that we saw them once a day; the morning... we had to wait.”

**625**: “What I didn’t expect to receive was the dietician care, I thought they would stop my HG problem and send me home, but they kept me after that for 5 days, and the dietician came every morning and evening to check and she gradually introduced [food] into my system and I’m perfect. By the time I left the hospital, I was taking everything, started eating everything”

In contrast, in many cases, there was a notable lack of connection made between the patient and their doctor:

**619:** "The nutritionist was very good … she helped me out too, she seemed to be the main caregiver, I didn’t really see the medical students or the residents or the doctor ever … it was really the dietician that came in every day to see me. And I really got a lot of support from the dietician, emotional support, and talk in general about what to expect down the road, and they were very good there."

## Discussion

HG symptoms caused distress in the mothers of our sample by severely hindering their abilities to keep up with their daily routines, and by leaving them feeling powerless, anxious, isolated, and hopeless. These results are consistent with previous studies that identify psychological distress as a reaction to somatic symptoms of HG [24–27]. However, this does not exclude that HG symptoms may have been exacerbated by high levels of distress, which, affected 40% of the mothers in our sample. As immigrant women described experiencing significant stress adapting to their new country, these findings are in line with a previous study showing that climate, changes in food, and loss of social support were among stress-inducing factors that South Asian immigrant women cited as having compromised their mental health [30]. However, the few women scoring the highest on the EPDS scale had been living in Canada for the longest, thus suggesting that other factors may have been responsible for their high level of distress. Our findings thus correspond to a survey that suggests that immigrants staying in Canada for a longer period of time tend to lose the healthy immigrant effect by presenting with lower mental health scores [31]. Immigrant women of our sample suffered substantially from the absence of their family members, particularly their mothers or sisters, thus adding to their level of distress. This corresponds to the literature suggesting that family support plays a key role during pregnancy in most cultures and communities [43].

### Accessibility to care

Many patients experienced long waiting times in emergency rooms, and in some cases were left undiagnosed for an extended period of time because their symptoms were misinterpreted by staff as being part of ordinary pregnancy. This lack of clinical attention is particularly concerning because HG leads to severe dehydration, which can be fatal or cause permanent harm to both the mother and the unborn child [44]. These include acute renal failure and encephalopathy in the mother [45, 46] and neurodevelopmental delay [47] and increased risk of psychological and behavioural disorders in adulthood in the child [17]. Direct referrals through the patients’ obstetricians would thus provide a better pathway to hospital care by reducing the risk of misdiagnosis and delayed treatment.

### Clinical alliance

A strong relationship between patients and their dieticians was noted, although comparable patient-physician relationships were noticeably lacking. We speculate that dieticians were probably most appreciated by mothers because they were able to directly address the immediate issue at hand (nutrition) by recommending ways to alleviate the mothers’ physical suffering and providing day-to-day support.

### Cultural meaning of HG

A salient result of this study is the fact that immigrant pregnant women could not link their debilitating symptoms to any cultural explanatory model or attribution. However, three out of four Canadian-born women interviewed relied on a biomedical type of explanatory model by stating that their HG was caused by hormones, stress, and anxiety. To our knowledge, this is the first empirical study using the MINI that has found no cultural attribution to an illness involving serious or debilitating symptoms. This absence of cultural explanation, we argue, contributed to a “victim blaming” phenomenon expressed by the immigrant participants.

Victim blaming is known to occur when the victim of a crime or anything wrongful is held entirely or partially responsible for the harm done to them. Psychologists believe that our tendency to blame the victim may originate, paradoxically, in a deep need to believe that the world is a good and just place in which “people get what they deserve” [48]. Our tendency to blame the victim is thus ultimately self-protective since it allows us to maintain an ideal worldview and to reassure ourselves that nothing bad will happen to us. Building from victimology theory [49], blaming mothers for their HG illness may be explained in three ways.

First, blaming mothers that suffer from HG may serve to give meaning to a debilitating illness that holds no clear biomedical or cultural explanation or attribution and thus is difficult to make sense of. Also, HG is a rare illness that can be confounded with the normal nausea and vomiting that affect pregnant women worldwide, thus adding to the confusion and need to maintain an “ideal worldview”.

Second, In the case of HG, there is no clear meaning available—whether biomedical or cultural—to explain why a woman cannot physically retain food while pregnant. This absurdity has important implications since, transculturally, pregnant women are generally expected to comply with behavioural and nutritional norms and practices that are widely shared with members of their family and cultural communities [50]. Thus family and community members commonly feel entitled to comment and give advice to pregnant women about their pregnancy, perhaps in an effort to safeguard the reproduction process that basically assures the survival of the community [50]. This may contribute to explain why family members and loved ones felt entitled to blame mothers who were unable to “keep food inside.”

Third, victimology theory [49] posits that a higher endorsement of binding values, or collectivist culture, reliably predicts stigmatising attitudes toward victims, while people who favour individualist values are more likely to be sympathetic toward victims. Indeed, in the case of this study, Canadian-born women with HG, who are from a Western individualist culture, did not report an experience of victim blaming by their loved ones. In contrast, many migrant families tended to perceive mothers as being partially responsible for their symptoms.

Building on the study by Mishel [51], which argues that the inability to attribute meaning to illness-related events can lead to increased psychological distress [51], we posit that the actual etiological uncertainty surrounding HG symptoms may have added to the immigrant women’s suffering. Adding to previous reports that associated HG with adverse effects on partner relationships and psychosocial distress [52], a tendency to restrict daily activities, and elevated stress and anxiety [27], our study points to an exacerbation of stress and anxiety in immigrant women suffering from HG stemming not only from the stress of adaptation to a new country while being pregnant but also from their “victim blaming” experience and the absence of key female family members.

### Clinical implications

Participants remarked that when they received a formal diagnosis from their physician they were reassured by the news that their symptoms were caused by a recognized illness even if they could not explain why they had HG. A medical diagnosis can validate patients’ experience, convey the legitimacy of their suffering to others, guide treatment, and mobilize resources. Clearly, an increase in the availability and accessibility of public information on HG is necessary. We suggest developing Web-based resources, since the Internet is the most popular source of health information [53]. In addition, having family present when medical staff explain the illness to mothers could convey its seriousness and prevent family members from interpreting the patient’s suffering as being exaggerated.

### Limitations and future research

The presence of husbands during a few interviews may have hindered participants’ disclosure of more serious sources of distress, such a family violence or other types of psychosocial problems. Our sample is multicultural and thus adds to theoretical generalisation, but remains a small qualitative study in one geographic location and as such may not be generalizable to all other settings. The small size of our control sample also suggests the need for further studies conducted on the meaning and experience of HG with larger samples of Canadian-born women, which could be compared to a larger sample of immigrant women.

## Conclusions

While the symptoms of HG do create distress in all women, our results suggest that migration stress, loss of family support, and the experience of “victim blaming” are important contributors to the high level of distress experienced by the immigrant pregnant women. Wider awareness of the impact of hyperemesis gravidarum may also improve the quality of family and social support, as well as accessibility and appropriateness of clinical care.

## Data Availability

Not applicable.

## Abbreviations

EPDS: Edinburgh Postnatal Depression Scale
HG: Hyperemesis Gravidarum
MINI: McGill Illness Narrative Interview Schedule
QCA: Qualitative Comparative Analysis Design
